# Plasma Cell Neoplasms: Genetics, Pathobiology, and New Therapeutic Strategies

**DOI:** 10.1155/2014/941820

**Published:** 2014-12-28

**Authors:** Dong Soon Lee, Wee-Joo Chng, Kazuyuki Shimizu

**Affiliations:** ^1^Department of Laboratory Medicine, Seoul National University College of Medicine, Seoul 110-744, Republic of Korea; ^2^Cancer Science Institute of Singapore, NUS, Singapore; ^3^Department of Hematology, Tokai Central Hospital, Japan

Plasma cell neoplasms are phenotypically heterogeneous disorder with broad spectrum, including plasma cell myeloma, monoclonal gammopathy of unknown significance, and amyloidosis. Each plasma cell neoplasm is defined by diagnostic criteria and, accordingly, therapeutic strategies are developed. Scientists often have difficulties in discovering genetics of plasma cell neoplasms due to problem of sorting plasma cells. Deep sequencing utilizing next generation sequencing or single cell genomics are expected to break through this barrier, providing novel tools to characterize the plasma cell neoplasms.

In this special issue on plasma cell neoplasms, the accepted articles can be divided into 4 categories: (i) genetic features of plasma cell myeloma, (ii) new prognostic factors, (iii) new therapeutic strategies, and (iv) features of Waldenstrom's macroglobulinemia.

*(i) Genetic Features of Plasma Cell Myeloma*. W.-J. Chng et al. reviewed changes of microRNA,* p53* gene, and MMSET in myeloma. They specially focused on the evidences of multilayer heterogeneity coexisting in the same patient, clinical parameters, genetic level, cell differentiation level, and clonal heterogeneity. Revealing this heterogeneity will break through challenges in terms of treatment, prognostication, and monitoring of treatment. Y. Koh et al. described the establishment of 2 cell lines from both myeloma bone marrow and plasmacytoma from a single patient and, interestingly, their biologic natures from one patient were different, which also shows a slice of the heterogeneity of myeloma. A multicenter cytogenetic study using Agilent 44 K aCGH microarrays in Czech also showed large genomic heterogeneity in MM cases and revealed copy number alterations in almost all cases. 

*(ii) New Prognostic Factors*. Many prognostic parameters were suggested in each special situation such as before peripheral stem cell mobilization, in infected patients, in patients with bone involvement, in complete remission group, in patients without chromosome abnormalities, and in elderly patients. Impact of RBC index, expression of myeloid antigens on plasma cells, plasma cell assessment before peripheral blood stem cell mobilization, impact of comorbidity, serum parathyroid hormone, hyperglycemia, and plasma level of osteopontin on clinical outcome were presented. C.-K. Min et al. suggested a significant impact of <5% BMPCs in patients who did not achieve immunofixation electrophoresis negativity and in patients with myeloma undergoing autologous stem cell transplantation. 

*(iii) New Therapeutic Strategies*. K. Shimizu et al. suggested that ASCT could also be a mainstay in the initial treatment of elderly MM patients, and its indication should be evaluated based on performance status and the presence of complications and/or comorbidities of each elderly patient with MM. H. J. Kim et al. suggested an alternative one-hour IV infusion mode of injection after evaluation of the response rate and incidence of bortezomib induced peripheral neuropathy. F. Raimondo summarized new insights into salvage therapy for relapsed/refractory MM as emerging from recent clinical trials exploring the activity of bendamustine, new generation proteasome inhibitors, novel IMiDs, monoclonal antibodies, and drugs interfering with growth pathways.

*(iv) Waldenstrom's Macroglobulinemia*. H. S. Lee et al. reported the higher overall response rate in patients treated with novel agent combined chemotherapy, compared to conventional chemotherapy alone, and in patients with Waldenstrom's macroglobulinemia. S. Y. Kim et al. reported an association of MYD88 L265P mutation and the presence of 6q deletion.

*Dong Soon Lee* *Wee-Joo Chng* *Kazuyuki Shimizu*

